# LncRNAs: The Regulator of Glucose and Lipid Metabolism in Tumor Cells

**DOI:** 10.3389/fonc.2019.01099

**Published:** 2019-11-26

**Authors:** Wei Lu, Fenghua Cao, Shengjun Wang, Xiumei Sheng, Jie Ma

**Affiliations:** ^1^Department of Immunology, Jiangsu Key Laboratory of Laboratory Medicine, School of Medicine, Jiangsu University, Zhenjiang, China; ^2^Zhenjiang Hospital of Chinese Traditional and Western Medicine, Zhenjiang, China

**Keywords:** lncRNAs, tumor, glucose, lipid, metabolism, therapy

## Abstract

Metabolism is a complex network of regulatory system. Cells often alter their metabolism in response to the changes in their environment. These adaptive changes are particularly pronounced in tumor cells, known as metabolic reprogramming. Metabolic reprogramming is considered to be one of the top 10 characteristics of tumor cells. Glucose and lipid metabolism are important components of metabolic reprogramming. A large number of experimental studies have shown that long non-coding RNAs (lncRNAs) play an important role in glucose and lipid metabolism. The current review briefly introduces the regulatory effect of lncRNAs on glucose and lipid metabolism of tumor cells, and the significance of lncRNA-mediated metabolism in tumor therapy, hoping to provide new strategies for clinical targeting tumor therapy.

## Introduction

Long non-coding RNAs (lncRNAs) are a class of RNA molecules <200 bp in length. LncRNAs are normally transcribed by RNA polymerase II and undergoes 5′ end capping, RNA splicing, and polyadenylation procedures (1, 2). LncRNAs do not code or have very low protein-coding capacity (3), and they have been regarded as “transcriptional noise” without any biological effects in the past (4). However, increasing evidence shows that lncRNAs are involved in gene regulation (5), cell cycle regulation (6), cell differentiation (7), immune response (8), tumor metabolism (9), and other processes (10, 11). LncRNAs often perform different functions in the cytoplasm and the nucleus (2). Interestingly, environmental transitions or infections can also induce changes of the lncRNA localization in the nucleus and the cytoplasm. For example, the nuclear export and mitochondrial localization of lncRNA RMRP is regulated by G-rich RNA sequence-binding factor 1 and human antigen R in the environments (12). In general, the molecular mechanisms of lncRNAs mainly include signals, decoys, guides, and scaffolds (13). As signal molecules, lncRNAs could mark the regulation of space, time, developmental stage, and gene expression. A signal molecule, linc-p21 as an example, is found to play an important role in apoptotic response after DNA damage, and this depends on the p53 pathway (14). In addition, lnc-c/EBPβ and lnc-chop are found to regulate the immunosuppressive function of myeloid-derived suppressor cells (MDSCs) in tumor and inflammatory environments, which as scaffolds bring together multiple proteins to form complexes (15, 16).

In the tumor microenvironment, lncRNAs may show oncogenic or tumor-suppressive functions due to the changes in its expression (17). The hypoxia, low pH, and energy stress of the tumor microenvironment are all factors that cause alteration in lncRNAs. It is reported that energy stress-induced lncRNA FILNC1 and lncRNA HAND2-AS1 could repress tumor development by regulating energy metabolism (18, 19). Hypoxia is also one of the microenvironmental cues responsible for lncRNAs changes, for example, the overexpression of lncRNA EIF3J-AS1 in atocellular carcinoma and lncRNA-HAL in breast cancer (20, 21). HIF-1α and c-Myc are often involved in the regulation of lncRNAs under hypoxia conditions. The potential mechanisms of dysregulation of lncRNAs will not be described in detail; here, we will focus on the regulatory effects of lncRNAs on glucose and lipid metabolism.

It is commonly believed that the balance of glucose, fatty acid, and protein metabolism plays an essential role in mammals. Once this metabolic balance is broken, it may cause various diseases, even tumor (22). Abundant evidence reveals that during the process of various diseases, especially cell carcinogenesis (23), the metabolic pattern changes significantly, involving glycolysis, mitochondrial oxidative phosphorylation, fatty acid oxidation, and other aspects. The researchers called this phenomenon the metabolic reprogramming of tumor cells (24–27). Glucose metabolism and lipid metabolism are the main energy metabolism modes of organisms, and they are closely related. Moon et al. reported that the products of glucose metabolism are substrates of lipid synthesis. Androgen can stimulate the conversion between metabolisms, and it increases the utilization of glucose by activating HK2 and PFKFB2 to provide a sufficient carbon source for fatty acid synthesis (28). Researchers also detected that the lactic acid from active TCA cycle is the primary raw material for fatty acid synthesis in glioblastoma cells by using ^13^C NMR spectroscopy (29). It has been reported that in the tumor microenvironment, the majority of tumor cells choose to increase glycolysis to meet their own energy and material needs, but some prefer to fatty acids as an energy source. In adipocytes and breast cancer cell co-culture models, breast cancer cells prefer to use β-oxidation of fatty acids (FAO) to supply the energies for proliferation and migration, which may be related to the tissue specificity of the breast (30). There are also some reports that suggest that under metabolic pressure, tumor cells ingest fatty acids in the surroundings and perform FAO to provide nutrients when glucose levels are low (31). Also, the amount of ATP produced by the full oxidation of each fatty acid molecule is more than twice that of glucose (32).

In conclusion, the metabolic mode of tumor cells is complex and changeable, and under pathological conditions, tumor cells will choose the optimal metabolic mode for their survival according to the different environments in which they are located. Recently, with the development of metabolic reprogramming of tumor cells, the roles of lncRNAs in glucose and lipid metabolism become a research hotspot, especially in glucose metabolism. Numerous evidence shows that lncRNAs can reprogram glucose and lipid metabolism in tumor cells, which influence tumor initiation, development, and progression, and may serve as a promising novel target for diagnosis and treatment of tumor.

## LncRNAs Participate in Glucose Metabolism in Tumor Cells

As the most important energy source of tumor cells, glucose metabolism mainly includes glucose uptake (primary entrance), entry into mitochondrial oxidative phosphorylation, and excretion of lactate (two primary exits) (33). Also, a little bit goes into pentose phosphate pathway (PPP). Glucose metabolic reprogramming is an important feature of tumor cells, providing sufficient ATP and NADPH for tumor cells to adapt to the changes in survival conditions and rapid proliferation. Under normal physiological conditions, mitochondrial oxidative phosphorylation is the main source of ATP, while tumor cells tend to have enhanced glycolysis even under sufficient oxygen conditions (34–36). Compared to oxidative phosphorylation, which produces 36 molecules of ATP, glycolysis only produces two molecules of ATP. However, ATP is formed faster than oxidative phosphorylation and it provides abundant macromolecular precursors to meet the demands for rapid growth and differentiation of tumor cells to the maximum extent. This is known as Warburg effect (37). In addition, the PPP is also an important process for the synthesis of biomacromolecules and reduction of equivalent (38). In the process of metabolic reprogramming, glucose transporters and many key enzymes determine the metabolic rate of glucose in most cases. The expression levels of these dominating enzymes usually markedly change during cell carcinogenesis (27). It is reported that lncRNAs regulate glucose metabolism primarily by regulating key enzymes in the glucose metabolism pathway.

### LncRNAs Participate in Glucose Uptake

**Figure 1:**
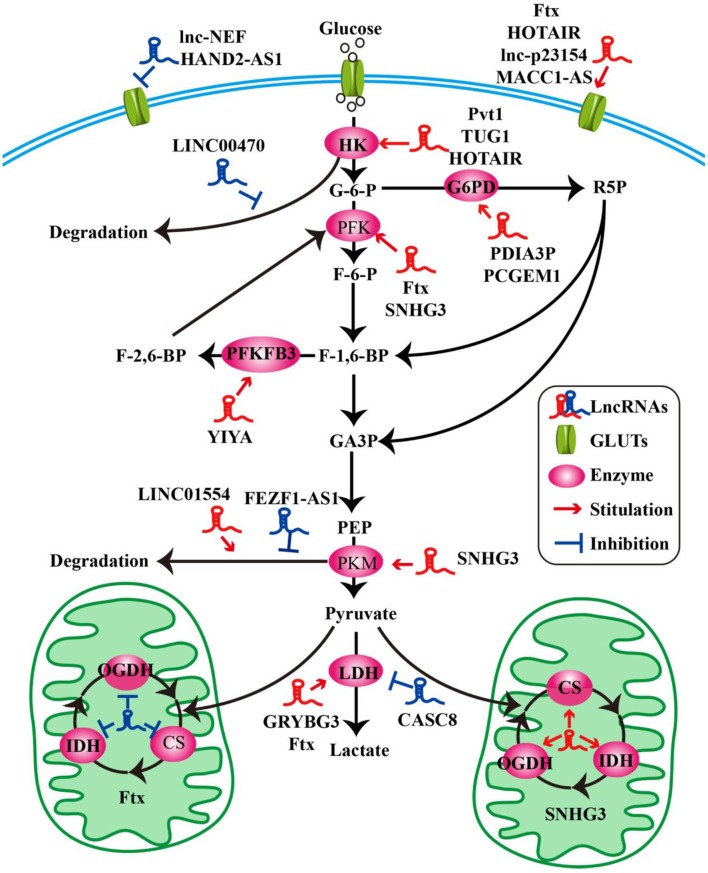
LncRNAs modulate the molecules of glucose metabolism in tumor cells. LncRNAs modulate glucose uptake by GLUTs. LncRNAs influence glycolysis, oxidative phosphorylation, and pentose phosphate pathway by key enzymes.

Glucose transporters (GLUTs) are a family of transmembrane protein that regulate the entry of extracellular glucose into cells during the processes of glucose metabolism (39). There are many subtypes of GLUTs, of which GLUT4, GLUT3, GLUT2, and GLUT1 are mostly relevant to the glucose metabolism. Insulin-regulated GLUT4 membrane transport is the most important glucose transporter in adipocytes and skeletal muscle cells (40). GLUT3 is a kind of glucose transporter mainly distributed in nerve tissue (41). GLUT2 is critical for the maintenance of hepatocytes' glucose balance and meanwhile plays a key role in the glucose perception of the nervous system (42). GLUT1 is a kind of glucose transporter that is widely distributed in many tissues and organs (43). It regulates basal glucose uptake during maintenance of normal physiological functions of most cells. This may be closely related to the rapid growth of tumor cells requiring a large amount of glucose. The expression and dysfunction of GLUTs are related to many diseases, especially in the metabolism of tumor cells (44). For example, the deficiency of lncRNA HAND2-AS1 is identified to up-regulate GLUT1 and GLUT3 in the process of promoting glucose uptake in osteosarcoma (19) (Figure 1 and Table 1).

**Table 1:** LncRNAs involve in glucose metabolism.

**lncRNAs**	**Metabolism-related enzyme**	**Tumor/cell types**	**Year of publication**	**References**
HAND2-AS1	GLUT1, GLUT3	Osteosarcoma	2018	(19)
lncRNA Ftx	GLUT1, GLUT4 PFK, LDH, CS IDH, OGDH	Hepatocellular carcinoma	2018	(45)
lnc-p23154	GLUT1	Oral squamous cell carcinoma	2018	(46)
lncRNA-NEF	GLUT1	Non-small cell lung cancer cells	2019	(47)
HOTAIR	GLUT1	Hepatocellular carcinoma	2017	(48)
MACC1-AS1	GLUT1	Gastric cancer	2018	(49)
SNHG3	PFK, PKM, CS IDH, OGDH	Ovarian cancer	2018	(50)
LINC00470	HK1	glioblastoma	2018	(51)
TUG1	HK2	Hepatocellular carcinoma	2018	(52)
TUG1	HK2	Osteosarcoma	2018	(53)
Pvt1	HK2	Osteosarcoma	2017	(54)
Pvt1	HK2	Gallbladder cancer	2019	(55)
HOTAIR	HK2	Oesophageal squamous cell carcinoma	2017	(56)
YIYA	PFKFB3	Breast cancer	2018	(57)
FEZF1-AS1	PKM2	Colorectal cancer	2018	(58)
LINC01554	PKM2	Hepatocellular carcinoma	2019	(59)
CRYBG3	LDHA	Lung cancer	2018	(60)
CASC8	LDHA	Bladder cancer	2017	(61)
PDIA3P	G6PD	Multiple myeloma	2018	(62)
PCGEM1	G6PD	Prostate cancer	2014	(63)

In pathological conditions, impaired membrane transport or functions of certain GLUTs are an important cause of disorders of glucose levels in various tumor cells, such as hepatocellular carcinoma (HCC) cells (45, 48), renal tumor cells (64), osteosarcoma cells (65), and so on. The high expression of GLUTs satisfies the energy needs of tumor cells. In a recent study, a new lncRNA named lnc-p23154 was found to promote glucose uptake and glycolysis by GLUT1, and it affected oral squamous cell carcinoma metastasis and invasion. A further study demonstrated that lnc-p23154 inhibited the transcription of miR-378a-3p by interacting with the promoter of miR-378a-3p. MiR-378a-3p could then bind to the 3′UTR of GLUT1 directly and repressed GLUT1 expression both at the mRNA and at the protein level (46). In non-small-cell lung cancer (NSCLC), the lncRNA-NEF is down-regulated, comparing adjacent healthy tissues and tumor tissues in patients with NSCLC. The overexpression of lncRNA-NEF inhibits NSCLC cell proliferation and glucose uptake and down-regulated GLUT1 expression. In short, lncRNA-NEF targets GLUT1 to influence the proliferation of NSCLC cells (47). The relationship between glucose uptake and HOTAIR has also been revealed in HCC cells. HOTAIR mediates the expression of GLUT1 via activating mammalian target of rapamycin (mTOR) signaling, which may provide a therapeutic strategy for HCC (48) (Figure 1 and Table 1).

Besides influencing the expression of GLUT1, lncRNAs are also likely to affect the distribution of GLUT1 in tumor cells to regulate the uptake of glucose. In gastric cancer cells, the expression of GLUT1 surrounding the cell membrane is induced by MACC1-AS1, which is a lncRNA, that is highly expressed under metabolic pressure. This is an indication that MACC1-AS1 is likely to promote glucose uptake and then promote glycolysis by increasing the distribution of GLUT1 in the vicinity of the cell membrane. However, the specific relationship between the MACC1-AS1 and GLUT1 remains to be further studied (49). The above results indicate that the role of lncRNAs in glycolysis-mediated proliferation or metastasis of tumor cells depends on GLUTs (Figure 1 and Table 1).

### LncRNAs Participate in Glycolysis and Oxidative Phosphorylation

As we all know, the cells absorb glucose in the cytoplasm and catalyze the formation of pyruvate through a series of key enzymes of glucose metabolism. The process by which pyruvate enters the mitochondria to produce large amounts of energy is called mitochondrial oxidative phosphorylation, while the process in which pyruvate is oxidized directly in the cytoplasm to produce lactic acid that does not enter the mitochondria is called glycolysis. Glycolysis and mitochondrial oxidative phosphorylation are inseparable. The process of glycolysis and mitochondrial oxidative phosphorylation involve a large number of enzymes, of which the major ones include hexokinases (HKs) (66), pyruvate kinase enzyme M (PKM) (67), lactate dehydrogenase (LDH) (68), citrate synthase (CS), and so on. Alterations of lncRNAs drive tumor cells to aerobic glycolysis and mitochondrial oxidative phosphorylation through regulation of metabolic enzymes involving these pathways. In HCC progression, lncRNA Ftx affects glucose metabolism reprogramming through the PPARγ pathway. On the one hand, it promotes glycolysis by promoting the expression and activity of phosphofructokinase (PFK) and LDH, and at the same time weakens the activity and expression of tricarboxylic acid cycle key enzymes CS, isocitrate dehydrogenase (IDH), and α-ketoglutarate dehydrogenase (OGDH) (45). The lncRNA SNHG3 is likely to play a similar role. Li et al. found that SNHG3 could up-regulate the expression of the metabolic enzymes PFK, PKM, CS, IDH, and OGDH to regulate the energy metabolism of ovarian cancer through mitochondrial proteomics analysis (50) (Figure 1 and Table 1).

HKs catalyzes the first and irreversible step of glycolysis (69). Hexokinase 1 (HK1) is one of the subtypes of HK. A cytoplasmic lncRNA LINC00470 involving fused in sarcoma (Fus), AKT, and HK1 pathway promotes glycolysis in glioblastoma cells by repressing HK1 ubiquitination (51). In addition to HK1, there are three other enzymes of HK identified, out of which hexokinase 2 (HK2) is the major enzyme that is closely involved in tumor cell glycolysis (70). For example, lncRNA taurine up-regulated gene 1 (TUG1) is disclosed to be related to HK2 involved in glycolytic metabolism and cell metastasis in HCC. The process involves a series of cascades, including TUG1/p21/miR-455-3p/AMPKβ2/HK2/Snail (52). TUG1 has also been associated with the HK2-mediating glycolysis regulating the viability ability of osteosarcoma cells, although the exact mechanism is unknown (53). In addition, one of the main functions of lncRNAs is to interact with microRNAs acting as a molecular sponge. It has been reported that lncRNA Pvt1, which is usually regarded as an oncogene, influences the expression of HK2, promoting glycolysis, and tumor progression in osteosarcoma and gallbladder cancer through acting as a competitive endogenous RNA to directly bind to miR-497 or miR-143, respectively (54, 55). Similarly, HOTAIR binding to miR-125 and miR-143 directly promotes the expression HK2 in esophageal squamous cell carcinoma cells (56). The above research findings fully show that HK2 is implicated in the regulation of tumor progression and metabolic programs involving lncRNAs (Figure 1 and Table 1).

PFKFB3 and PKM2 are also key enzymes regulating glycolysis and oxidative phosphorylation. LncRNA YIYA regulates the PFKFB3 phosphorylation in cyclin-dependent kinase 6-dependent pathway, promoting glycolysis in breast cancer cell (57). The overexpression of lncRNA FEZF1-AS1 in colorectal cancer cells reduced the ubiquitination degradation of PKM2. As a result, the expression of cytoplasmic and nuclear PKM2 protein in glucose metabolism process increased (58). Contrary to FEZF1-AS1, LINC01554 shows a suppressive function because of the acceleration in PKM2 ubiquitination degradation in HCC cells (59) (Figure 1 and Table 1).

LDHA catalyzes the last step of aerobic glycolysis, which is highly critical to the glycolysis phenotype of tumor cells (71). Overexpression of LDHA promotes tumor cell malignant transformation and growth, indicating the important role of LDHA in tumor initiation or maintenance (68). In lung cancer cell lines, it is reported that lncRNA CRYBG3 regulates glycolysis rather than oxidative phosphorylation to increase lung cancer cell proliferation through interacting with LDHA. Knockdown of lncRNA CRYBG3 reduces the expression and activity of LDHA and then decreases the consumption of glucose and pyruvate; however, there are no notable changes in the level of oxidative phosphorylation including all kinds of tricarboxylic acid cycle intermediates regardless of overexpression or knockdown lncRNA CRYBG3 (60). In bladder cancer, the lncRNA cancer susceptibility candidate 8 (CASC8) is reported to function as a tumor suppressor and reduces glycolysis via inhibiting fibroblast growth factor receptor 1-mediated LDHA phosphorylation at Tyr10 (61) (Figure 1 and Table 1).

### LncRNAs Participate in the PPP

The PPP is another way of catabolizing glucose, which can produce large amounts of NADPH, providing a reducing agent for various synthesis reactions of tumor cells. The ribose-5-phosphate (R5P) produced by this pathway can also provide raw materials for the synthesis of many substances (72). Glucose-6-phosphate dehydrogenase (G6PD) is the first key enzyme in the oxidation phase of the PPP, which transfers glucose-6-phosphate to 6-phosphogluconate. G6PD is up-regulated in many tumor cells and tumor tissues and generally its level correlates with the overall survival of patients. The lncRNA protein disulfide isomerase family A member 3 pseudogene 1 (PDIA3P) is a 2099-bp lncRNA located at chromosome 1q21.1. It has been reported that PDIA3P regulates multiple myeloma growth and drug resistance through up-regulating G6PD in the PPP. Researchers further discovered that PDIA3P increased the G6PD expression and the PPP flux through enhancing c-MYC transactivation activity bound to the G6PD promoter (62). Similar to PDIA3P, the lncRNA prostate cancer gene expression marker 1 (PCGEM1) also affects pentose phosphate shunt to facilitate biosynthesis of nucleotide and generates NADPH for redox homeostasis as a coactivator for c-MYC and androgen receptor (63). Although the PPP is not the main pathway of glucose metabolism and there are not many studies on lncRNAs and PPP, it is reasonable to believe that PPP is also an important link in the regulation of lncRNAs during tumor progression (Figure 1 and Table 1).

## LncRNAs Participate in Lipid Metabolism in Tumor Cells

**Figure 2:**
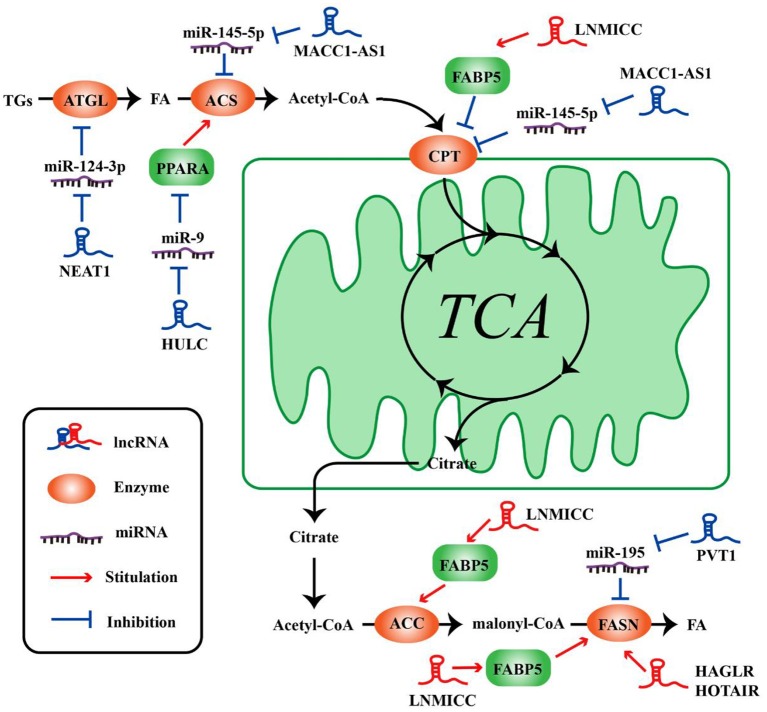
LncRNAs regulate the molecules of lipid metabolism in tumor cells. LncRNAs regulate lipid synthesis and decomposition by different ways and molecules.

The metabolism of tumor cells is completely different from that of normal cells; in addition to glucose metabolism, lipid metabolism may also undergo adaptive changes (73, 74). Such changes in the overall metabolic pattern constitute the metabolic reprogramming of tumor cells (27). Lipid biosynthesis is not surprising as a part of metabolic abnormalities in tumor cells, which require a large amount of lipid to synthesize biofilms, organelles, and important signaling molecules during rapid proliferation (32). As such, FAO also could provide ATP for tumor cells (31), and one molecule of fatty acid produces much more ATP than glucose. Numerous key enzymes related to lipid synthesis and lipolysis are highly expressed in tumor cells. LncRNAs can be involved in the regulation of multiple lipid metabolism-related genes in tumor cells. It is reported that lncRNA associated with lymph node metastasis in cervical cancer (LNMICC) facilitates fatty acid metabolism reprogramming to promote lymph node metastasis of cervical cancer cells by regulating fatty acid-binding protein 5 (FABP5), which is a carrier of fatty acid uptake and transport. Many key genes involved in fatty acid metabolism, including fatty acid synthase (FASN), carnitine palmitoyl transferase 1 A (CPT1A), acyl-CoA oxidase 1 (ACOX1), and acetyl-CoA carboxylase 1 (ACC1), are all altered by the gain- and loss-of-function strategies of LNMICC, and this regulation of LNMICC on lipid metabolism depends on FABP5 (75) (Figure 2 and Table 2).

**Table 2:** LncRNAs involve in lipid metabolism.

**lncRNAs**	**Metabolism-related gene**	**Tumor types**	**Year of publication**	**References**
HULC	ACSL1	Hepatocellular carcinoma	2015	(76)
LNMICC	FASN, ACC1 ACOX1, CPT1	Cervical cancer	2017	(75)
LINC01138	SREBP1	Clear cell renal cell carcinoma	2018	(77)
HAGLR	FASN	Non-small cell lung cancer	2017	(78)
HOTAIR	FASN	Human nasopharyngeal carcinoma	2017	(79)
Pvt1	FASN	Osteosarc**o**ma	2016	(80)
NEAT1	ATGL	Hepatocellular carcinoma	2018	(81)
MACC1-AS1	ACS, CPT1A	Gastric cancer	2018	(82)

Lipid metabolism balance includes lipid uptake, synthesis, catabolism, and secretion. Currently, abundant lncRNAs have been confirmed to be involved in the regulation of lipid metabolism (83, 84). However, lncRNAs involved in lipid metabolism is mostly associated with cardiovascular disease or hepatic disease. There are few reports on the lipid metabolism functions of lncRNAs in tumor cells. The following headlines below principally discusses the role of lncRNAs in lipid synthesis and lipolysis.

### LncRNAs Participate in Lipid Synthesis

Lipid synthesis is a landmark change in tumor cells. Synthesis of lipid can not only provide a large number of membrane phospholipids as raw materials for the continuous division and proliferation of tumor cells, but also synthesize a series of cancer-promoting lipid signaling molecules, such as phosphatidylinositol and sphingomyelin, etc.

FASN is a key rate-limiting enzyme catalyzing *de novo* fatty acid biogenesis (85). Since FASN was discovered, its role in tumor growth and intracellular signal transduction has been widely studied. HAGLR, also called HOXD-AS1, is generally elevated in NSCLC and it is regarded as a predictor of poor patient survival. The proliferation and invasion ability of NSCLC cells decreased after HAGLR knockdown and cells mainly remained in the G1 phase. Furthermore, the expression of FASN and the amount of free fatty acids significantly reduced with the decrease of HAGLR expression in the non-small cells (78). In human nasopharyngeal carcinoma cells, high expression of HOTAIR is positively correlated with FASN expression. Adenovirus encoded short hairpin RNA knockdown of HOTAIR causes the decrease of free fatty acid and FASN at transcriptional and post-transcriptional levels in nasopharyngeal carcinoma cells (79). Although the specific mechanism, of how lncRNAs regulate lipid synthesis involving FASN is unclear, they at least partly demonstrate that lncRNAs play an important role in tumor cells by FASN-mediated lipid synthesis. In osteosarcoma cells, lncRNA Pvt1 was found to act as a sponge molecule to adsorb miR-195. As a result, the expression of FASN, B-cell lymphoma 2 protein, and Cyclin D1 increased. At the same time, the study also showed that silencing Pvt1 reduced cell invasion, and FASN could reverse this effect (80). Sterol regulatory element binding protein 1 (SREBP1) is a major transcription factor involved in lipid accumulation and desaturation. Zhang et al. in their study indicated that the lncRNA LINC01138 increased the arginine methylation of SREBP1 at the post-transcriptional level to regulate lipid desaturation and cells growth of clear cell renal cell carcinoma by interacting with protein arginine methyltransferase 5 (77) (Figure 2 and Table 2).

### LncRNAs Participate in Lipolysis

Fat mobilization is the first step in lipid catabolism, and adipose triglyceride lipase (ATGL) is a first key lipase that has been discovered in recent years to initiate fat mobilization (86, 87). It is vital to maintain the balance of lipid storage mobilization (88). In fact, ATGL has been reported to be reduced in leiomyosarcoma, non-small cell lung cancer as well as pancreatic adenocarcinoma, and the levels of ATGL are associated with patients survival (89). In the diet-induced murine HCC model of steatohepatitis and human HCC, the expression of ATGL is also found to be decreased by Di Leo (90). Although it is frequently reported that ATGL shows anti-neoplastic effect, some studies have proposed its tumor-promoting function (91). Interestingly, Liu et al. found that the expression of ATGL at transcriptional level and post-transcriptional level is higher in human HCC tissues and orthotopic HCC mouse model, which is exactly the opposite of Di Leo. It is showed that high expression of ATGL promoted the growth of HCC cells by catalyzing the production of diacylglycerol (DAG) and free fatty acids (FFA). This opposite phenomenon may be due to differences in the clinicopathological features of the patients and the mouse model. Simultaneously, the study also indicated that the lncRNA NEAT1 could regulate the expression of ATGL and affect the abnormal lipidosis of HCC cells through ATGL. Further research confirmed miR-124-3p/ATGL/DAG+FFA to participate in the regulation effect of NEAT1 on the lipid decomposition in HCC and thus promoted the progress of HCC (92) (Figure 2 and Table 2).

It is well-known that the most important form of fatty acid decomposition is β-oxidation of fatty acids (FAO), and studies have shown that lncRNAs are also extremely important in the oxidation of fatty acids. Acyl-CoA synthetase (ACS) is the rate-limiting enzyme in the first step of the fatty acid oxidation reaction. HULC is the first lncRNA identified to be specifically elevated in HCC compared with normal liver tissues (93). Cui et al. revealed that, in hepatoma carcinoma cells, HULC could modulate lipogenesis by the pathway involving miR-9, peroxisome proliferator-activated receptor alpha (PPARA), and ACS long-chain family members 1 (ACSL1), and interestingly, ACSL1 also seems to have a positive feedback effect on HULC expression during this process (81). In gastric cancer cells, transforming growth factor β1 secreted by mesenchymal stem cells could promote the high expression of lncRNA MACC1-AS1. The high expression of MACC1-AS1 relieved the inhibitory effect of miR-145-5p on FAO by inhibiting miR-145-5p, ultimately promoting the stemness and chemoresistance depending on FAO of gastric cancer cells (94). Taken together, lncRNAs participate in lipid decomposition in different ways to affect tumor progress (Figure 2 and Table 2).

## LncRNAs Participate in the Adjuvant Therapy of Tumor Possibly

Tumor is the leading killer of human health. Luo et al. demonstrated that lncRNA MALAT1 was crucial in arsenite-induced hepatotoxicity. In the process of cell carcinogenesis induced by arsenic, there is also a phenomenon of enhanced glycolysis, which is mainly manifested by the accumulation of lactate, the acceleration of glucose consumption, and the increased expression of a series of glycolytic related genes, such as HK2, Eno1, and GLUT4. MALAT1 further stabilizes HIF-1α, enhancing the arsenite-induced glycolysis process by disrupting the VHL–HIF-1α interaction (76). It provides new insight into the mechanism of arsenic toxicity on the human body. For a long time, people have been committed to exploring and studying the mechanisms of tumor occurrence and development, trying to find a more effective therapy for tumors. LncRNAs are abnormally expressed in many tumors, and lncRNA-mediated metabolic reprogramming plays a key role in promoting and maintaining tumor formation and progression. These have shown the clinical potential as a therapeutic target of lncRNAs.

Radiotherapy is a common method of tumor treatment. Li et al. unveiled the role of UCA1 in the radiotherapy of cervical cancer from the perspective of glucose metabolism and proposed UCA1 as a potential target for improving the efficacy of radiotherapy for cervical cancer. The radiosensitivity of cervical cancer cells was enhanced after treating with glycolysis inhibitor 2-DG. Knockdown of UCA1 contributed to reduce glucose consumption and lactic acid production by down-regulation of HK2 expression rather than GLUTs or PKM. The discovery of the UCA1/HK2/glycolysis pathway in the radiotherapy of cervical cancer provides a new method to improve the radiotherapy effect (82). Abnormal energy metabolism is a characteristic of tumor cells, and biguanides are often used as potential tumor therapy drugs because of their role in inhibiting the mitochondrial respiratory chain (95). Phenformin is a type of biguanides used in the treatment of tumor. In this treatment with phenformin, lncRNA NBR2 has been discovered to have a potential role in the adaptive response of tumor cells. Elevated NBR2 expression reduced the apoptosis of tumor cells induced by phenformin. As a result, the number of tumor cells increased (96). Thus, the role of lncRNA-mediated metabolic reprogramming in tumor therapy cannot be ignored. They can, therefore, be used as an adjunct to other therapies to treat tumors.

## Summary and Prospect

In recent years, the studies of lncRNAs on tumor cells' metabolic reprogramming have developed rapidly. Numerous reports have been published on the different lncRNAs that affect the proliferation, migration, invasion, and other pathological processes of tumor cells by regulating the energy metabolism. As an important part of tumor microenvironments, similar to tumor cells, there is also a phenomenon of metabolic reprogramming in immune cells (97). Immune cells of different states and different stages of differentiation show different metabolic phenotypes (98, 99). For example, naive T cells are primarily dependent on FAO and oxidative phosphorylation to maintain their quiescence (100), while activated T cells and effector T cells, including Th1, Th2, and Th17, have a similar metabolic pattern to tumor cells and mainly obtain energy by aerobic glycolysis (98). A recent study found that the expression of lncRNA Malat1 was significantly different in macrophages treated with LPS and IL-4, respectively. Knockdown of Malat1 promoted the M2 macrophage polarization induced by IL-4. After the use of mitochondrial pyruvate carriers inhibitor UK-5099 or mitochondrial oxidative phosphorylation complex 1 inhibitor, the oxidative phosphorylation of macrophages was prevented, and the effect of Malat1 knockdown on M2 macrophage polarization was also eliminated (101), thus suggesting that Malat1-mediated alternative activation of macrophages is dependent on the elevated mitochondrial oxidative phosphorylation.

Notwithstanding, the regulation of lncRNAs on immune cell metabolism has rarely been reported, but the regulation of lncRNAs on immune cells has been extensively studied. MDSCs are a group of heterogeneous cells with significant immunosuppressive activity derived from bone marrow. It abnormally expands during inflammation, infection, and tumor microenvironments (102). Previous experiments in our laboratory showed that lncRNA RUNXOR accelerates MDSC-mediated immunosuppression via targeting RUNX1 in lung cancer (103). Pvt1 regulates the immunosuppression activity of granulocytic myeloid-derived suppressor cells (G-MDSCs), which is a subgroup of MDSCs, and Pvt1 is up-regulated by HIF-1α under hypoxia conditions in tumor-bearing mice (104). In turn, different living environments and metabolic state will also affect the phenotype and functions of immune cells, making immune cells more suitable for their functional needs (105–107). Also, Hossain et al. revealed that the oxidation of fatty acids could induce the immunosuppressive function of MDSCs and ultimately promoted the progression of the tumor (108). In nasopharyngeal carcinoma cells, the promotion of latent membrane proteins 1 on MDSC expansion also depends on GLUT1-mediated glycolysis (109).

Therefore, it is reasonable to believe that lncRNAs is also likely to regulate the function of immune cells by regulating the metabolic reprogramming. Of course, this conclusion remains to be confirmed by more studies. In-depth discussion of the effect of lncRNAs on the metabolic function of tumor cells and immune cells will help to further understand the mechanism of tumor occurrence, development, and the function of immune cells in tumor microenvironments, and could provide new ideas and strategies for clinical diagnosis and anti-tumor therapy.

## Author Contributions

All authors listed have made a substantial, direct and intellectual contribution to the work, and approved it for publication.

### Conflict of Interest

The authors declare that the research was conducted in the absence of any commercial or financial relationships that could be construed as a potential conflict of interest.

## Abbreviations

lncRNAs: long non-coding RNAs
MDSCs: myeloid derived suppressor cells
FAO: β-oxidation of fatty acids
GLUTs: glucose transporters
HCC: hepatocellular carcinoma
NSCLC: non-small-cell lung cancer
HK: hexokinase
PK: pyruvate kinase isozyme
LDH: lactate dehydrogenase
CS: citrate synthase
PFK: phosphofructokinase
IDH: isocitrate dehydrogenase
OGDH: α-ketoglutarate dehydrogenase
TUG1: lncRNA taurine up-regulated gene 1
CASC8: the lncRNA cancer susceptibility candidate 8
PPP: pentose phosphate pathway
G6PD: glucose-6-phosphate dehydrogenase
PDIA3P: protein disulfide isomerase family A member 3 pseudogene 1
PCGEM1: long non-coding RNA prostate cancer gene expression marker 1
LNMICC: lncRNA associated with lymph node metastasis in cervical cancer
FABP5: fatty acid-binding protein 5
FASN: fatty acid synthase
CPT1A: carnitine palmitoyl transferase 1 A
ACOX1: acyl-CoA oxidase 1
ACC1: acetyl-CoA carboxylase 1
ACSL1: Acyl-CoA synthetase long-chain family members 1
SREBP1: sterol regulatory element binding protein 1
ATGL: adipose triacylglyceride lipase.
